# Nrf2-interacting nutrients and COVID-19: time for research to develop adaptation strategies

**DOI:** 10.1186/s13601-020-00362-7

**Published:** 2020-12-03

**Authors:** Jean Bousquet, Jean-Paul Cristol, Wienczyslawa Czarlewski, Josep M. Anto, Adrian Martineau, Tari Haahtela, Susana C. Fonseca, Guido Iaccarino, Hubert Blain, Alessandro Fiocchi, G. Walter Canonica, Joao A. Fonseca, Alain Vidal, Hak-Jong Choi, Hyun Ju Kim, Vincent Le Moing, Jacques Reynes, Aziz Sheikh, Cezmi A. Akdis, Torsten Zuberbier, the ARIA group, Amir Hamzah Abdul Latiff, Baharudin Abdullah, Werner Aberer, Nancy Abusada, Ian Adcock, Alejandro Afani, Ioana Agache, Xenofon Aggelidis, Jenifer Agustin, Cezmi A Akdis, Mübeccel Akdis, Mona Al-Ahmad, Abou Al-Zahab Bassam, Hussam Alburdan, Oscar Aldrey-Palacios, Emilio Alvarez Cuesta, Hiba Alwan Salman, Ashraf Alzaabi, Salma Amade, Gene Ambrocio, Rosana Angles, Isabella Annesi-Maesano, Ignacio J Ansotegui, Josep M. Anto, Paula Ara Bardajo, Stefania Arasi, Margarete Arrais, Hasan Arshad, Maria-Cristina Artesani, Estrella Asayag, Francesca Avolio, Khuzama Azhari, Claus Bachert, Diego Bagnasco, Ilaria Baiardini, Nissera Bajrović, Petros Bakakos, Sergio Bakeyala Mongono, Christine Balotro-Torres, Sergio Barba, Cristina Barbara, Elsa Barbosa, Bruno Barreto, Joan Bartra, Xavier Basagana, Eric D. Bateman, Lkhagvaa Battur, Anna Bedbrook, Martín Bedolla Barajas, Bianca Beghé, Antra Bekere, Elizabeth Bel, Ali Ben Kheder, Mikael Benson, Elena-Camelia Berghea, Karl-Christian Bergmann, Roberto Bernardini, David Bernstein, Mike Bewick, Slawomir Bialek, Artur Białoszewski, Thomas Bieber, Nils E. Billo, Maria-Beatrice Bilo, Carsten Bindslev-Jensen, Leif Bjermer, Hubert Blain, Irina Bobolea, Malgorzata Bochenska Marciniak, Christine Bond, Attilio Boner, Matteo Bonini, Sergio Bonini, Sinthia Bosnic-Anticevich, Isabelle Bosse, Sofia Botskariova, Jacques Bouchard, Louis-Philippe Boulet, Rodolphe Bourret, Philippe Bousquet, Fulvio Braido, Andrew Briggs, Christopher E Brightling, Jan Brozek, Luisa Brussino, Roland Buhl, Roxana Bumbacea, Rosalva Buquicchio, María-Teresa Burguete Cabañas, Andrew Bush, William W Busse, Jeroen Buters, Fernan Caballero-Fonseca, Moïses A Calderon, Mario Calvo, Paulo Camargos, Thierry Camuzat, FR Canevari, Antonio Cano, G. Walter Canonica, Arnaldo Capriles-Hulett, Luis Caraballo, Vicky Cardona, Kai-Hakon Carlsen, Jonas Carmona Pirez, Jorge Caro, Warner Carr, Pedro Carreiro-Martins, Fredelita Carreon-Asuncion, Ana-Maria Carriazo, Carme Carrion y Ribas, Thomas Casale, Mary-Ann Castor, Elizabeth Castro, A. G. Caviglia, Lorenzo Cecchi, Alfonso Cepeda Sarabia, Maciej Chalubinski, Ramanathan Chandrasekharan, Yoon-Seok Chang, Victoria Chato-Andeza, Lida Chatzi, Christina Chatzidaki, Niels H. Chavannes, Claudia Chaves Loureiro, Aurora-Alejandra Chavez Garcia, Marta Chelninska, Yuzhi Chen, Lei Cheng, Sharon Chinthrajah, Tomas Chivato, Ekaterine Chkhartishvili, George Christoff, Henry Chrystyn, Derek K Chu, Antonio Chua, Alexander Chuchalin, Kian Fan Chung, Alberto Cicerán, Cemal Cingi, Giorgio Ciprandi, Ieva Cirule, Ana-Carla Coelho, Enrico Compalati, Jannis Constantinidis, Jaime Correia de Sousa, Elisio Manuel Costa, David Costa, María del Carmen Costa Domínguez, André Coste, M. Cottini, Linda Cox, Carlos Crisci, Maria Angiola Crivellaro, Alvaro A Cruz, John Cullen, Adnan Custovic, Biljana Cvetkovski, Wienczyslawa Czarlewski, Gennaro D’Amato, Jane da Silva, Ronald Dahl, Sven-Erik Dahlen, Vasilis Daniilidis, Louei Darjazini Nahhas, Ulf Darsow, Janet Davies, Frédéric de Blay, Giulia De Feo, Eloisa De Guia, José-Ricardo De la Torre Navarrete, Chato De los Santos, Esteban De Manuel Keenoy, Govert De Vries, Diana Deleanu, Pascal Demoly, Judah Denburg, Philippe Devillier, Alain Didier, Sanja Dimic Janjic, Maria Dimou, Anh Tuan Dinh-Xuan, Ratko Djukanovic, Maria Do Ceu Texeira, Dejan Dokic, Margarita Gabriela Domínguez Silva, Habib Douagui, Nikolaos Douladiris, Maria Doulaptsi, Gérard Dray, Ruta Dubakiene, Eve Dupas, Stephen Durham, Marzia Duse, Mark Dykewicz, Didier Ebo, Natalija Edelbaher, Thomas Eiwegger, Patrik Eklund, Yehia El-Gamal, Zeinab A. El-Sayed, Shereen S. El-Sayed, Magda El-Seify, Regina Emuzyte, Lourdes Enecilla, Marina Erhola, Heidilita Espinoza, Jesús Guillermo Espinoza Contreras, John Farrell, Lenora Fernandez, Paola Fimbres Jimenez, Antje Fink Wagner, Alessandro Fiocchi, Wytske J Fokkens, Lenia Folletti, Joao A Fonseca, Jean-François Fontaine, Francesco Forastiere, Jose Miguel Fuentes Pèrez, Emily Gaerlan-Resureccion, Mina Gaga, José Luis Gálvez Romero, Amiran Gamkrelidze, Alexis Garcia, Cecilia Yvonne García Cobas, María de la Luz Hortensia García Cruz, Valeria Garcia Ortiz, Jacques Gayraud, Matteo Gelardi, Bilun Gemicioglu, Dimitra Gennimata, Sonya Genova, José Gereda, Roy Gerth van Wijk, Antonio Giuliano, René-Maximiliano Gomez, Miguel-Ange Gonzalez Ballester, Sandra González Diaz, Maia Gotua, Christos Grigoreas, Ineta Grisle, Marta Guidacci, Nick Guldemond, Zdenek Gutter, Antonieta Guzmán, Tari Haahtela, Ramsa Halloum, David Halpin, Eckard Hamelmann, Suleiman Hammadi, Richard Harvey, Enrico Heffler, Joachim Heinrich, Adnan Hejjaoui, Birthe Hellquist-Dahl, Luiana Hernández Velázquez, Mark Hew, Elham Hossny, Peter Howarth, Martin Hrubiško, Yunuen Rocío Huerta Villalobos, Marc Humbert, Salina Husain, Michael Hyland, Guido Iaccarino, Moustafa Ibrahim, Nataliya Ilina, Maddalena Illario, Cristoforo Incorvaia, Antonio Infantino, Carla Irani, Zhanat Ispayeva, Juan Carlos Ivancevich, Edgardo EJ Jares, Deborah Jarvis, Ewa Jassem, Klemen Jenko, Rubén Darío Jiméneracruz Uscanga, Sebastian L Johnston, Guy Joos, Maja Jošt, Kaja Julge, Ki-Suck Jung, Jocelyne Just, Marek Jutel, Igor Kaidashev, Omer Kalayci, Fuat Kalyoncu, Jeni Kapsali, Przemyslaw Kardas, Jussi Karjalainen, Carmela A. Kasala, Michael Katotomichelakis, Loreta Kavaliukaite, Kazi S. Bennoor, Thomas Keil, Paul Keith, Musa Khaitov, Nikolai Khaltaev, You-Young Kim, Bruce Kirenga, Jorg Kleine-Tebbe, Ludger Klimek, Fanny W. Ko, Bernard Koffi N’Goran, Evangelia Kompoti, Peter Kopač, Gerard Koppelman, Anja Koren Jeverica, Seppo Koskinen, Mitja Košnik, Tomasz Kostka, Kosta V. Kostov, Marek L Kowalski, Tanya Kralimarkova, Karmen Kramer Vrščaj, Helga Kraxner, Samo Kreft, Vicky Kritikos, Dmitry Kudlay, Mikael Kuitunen, Inger Kull, Piotr Kuna, Maciej Kupczyk, Violeta Kvedariene, Marialena Kyriakakou, Nika Lalek, Massimo Landi, Stephen Lane, Désiree E. Larenas-Linnemann, Susanne Lau, Daniel Laune, Jorge Lavrut, Lan Le, Martina Lenzenhuber, Gualtiero Leo, Marcus Lessa, Michael Levin, Jing Li, Philip Lieberman, Giuseppe Liotta, Brian Lipworth, Xuandao Liu, Rommel Lobo, Karin C Lodrup Carlsen, Carlo Lombardi, Renaud Louis, Stelios Loukidis, Olga Lourenço, Jorge A. Luna Pech, Bojan Madjar, Enrico Maggi, Antoine Magnan, Bassam Mahboub, Alpana Mair, Anke-Hilse Maitland van der Zee, Mika Makela, Michael Makris, Hans-Jorgen Malling, Mariana Mandajieva, Patrick Manning, Manolis Manousakis, Pavlos Maragoudakis, Gianluigi Marseglia, Gailen Marshall, Mohammad Reza Masjedi, Jorge F. Máspero, Juan José Matta Campos, Marcus Maurer, Sandra Mavale-Manuel, Cem Meço, Erik Melén, Giovanni Melioli, Elisabete Melo-Gomes, Eli O Meltzer, Enrica Menditto, Andrew Menzies-Gow, Hans Merk, Jean-Pierre Michel, Yann Micheli, Neven Miculinic, Luís Midão, Florin Mihaltan, Nikolaos Mikos, Manlio Milanese, Branislava Milenkovic, Dimitrios Mitsias, Bassem Moalla, Giuliana Moda, María Dolores Mogica Martínez, Yousser Mohammad, Frances-Montserrat Moharra, Mostafa Moin, Mathieu Molimard, Isabelle Momas, Monique Mommers, Alessandro Monaco, Stephen Montefort, Lucia-Elvira Montenegro, Riccardo Monti, Dory Mora, Mario Morais-Almeida, Ralph Mösges, Badr Eldin Mostafa, Joaquim Mullol, Lars Münter, Antonella Muraro, Ruth Murray, Antonio Musarra, Tihomir Mustakov, Robert Naclerio, Kari C. Nadeau, Rachel Nadif, Alla Nakonechna, Leyla Namazova-Baranova, Gretchen Navarro-Locsin, Hugo Neffen, Kristof Nekam, Angelos Neou, Eustachio Nettis, Daniel Neuberger, Laurent Nicod, Stefania Nicola, Verena Niederberger-Leppin, Marek Niedoszytko, Antonio Nieto, Ettore Novellino, Elizabete Nunes, Dieudonné Nyembue, Robyn E. O’Hehir, Cvetanka Odjakova, Ken Ohta, Yoshitaka Okamoto, Kimi Okubo, Brian Oliver, Gabrielle L Onorato, Maria Pia Orru, Solange Ouédraogo, Kampadilemba Ouoba, Francisco-Javier Padilla, Pier Luigi Paggiaro, Aris Pagkalos, Giovanni Pajno, Gianni Pala, SP Palaniappan, Isabella Pali-Schöll, Susanna Palkonen, Stephen Palmer, Carmen Panaitescu Bunu, Petr Panzner, Nikos G Papadopoulos, Vasilis Papanikolaou, Alberto Papi, Bojidar Paralchev, Giannis Paraskevopoulos, Hae-Sim Park, Giovanni Passalacqua, Vincenzo Patella, Ian Pavord, Ruby Pawankar, Soren Pedersen, Susete Peleve, Simona Pellegino, Ana Pereira, Mariana Pereira, Tamara Pérez, Andrea Perna, Diego Peroni, Oliver Pfaar, Nhân Pham-Thi, Bernard Pigearias, Isabelle Pin, Konstantina Piskou, Constantinos Pitsios, Davor Plavec, Dagmar Poethig, Wolfgang Pohl, Antonija Poplas Susic, Todor A. Popov, Fabienne Portejoie, Paul Potter, Lars Poulsen, Alexandra Prados-Torres, Fotis Prarros, David Price, Emmanuel Prokopakis, Francesca Puggioni, Elisa Puig-Domenech, Robert Puy, Klaus Rabe, Silvia Rabotti, Filip Raciborski, Josephine Ramos, Cristina Recalcati, Marysia T. Recto, Shereen M. Reda, Frederico S Regateiro, Norbert Reider, Sietze Reitsma, Susana Repka-Ramirez, Erminia Ridolo, Janet Rimmer, Daniela Rivero Yeverino, José Angelo Rizzo, Carlos Robalo-Cordeiro, Graham Roberts, Karen Robles, Nicolas Roche, Mónica Rodríguez González, Eréndira Rodríguez Zagal, Giovanni Rolla, Christine Rolland, Regina Roller-Wirnsberger, Miguel Roman Rodriguez, Antonino Romano, Jan Romantowski, Philippe Rombaux, Joel Romualdez, Jose Rosado-Pinto, Nelson Rosario, Lanny Rosenwasser, Oliviero Rossi, Menachem Rottem, Philip W. Rouadi, Nikoleta Rovina, Irma Rozman Sinur, Mauricio Ruiz, Lucy Tania Ruiz Segura, Dermot Ryan, Hironori Sagara, Daiki Sakai, Daiju Sakurai, Wafaa Saleh, Johanna Salimaki, Konstantinos Samitas, Boleslaw Samolinski, María Guadalupe Sánchez Coronel, Mario Sanchez-Borges, Jaime Sanchez-Lopez, Melissa Sansonna, Codrut Sarafoleanu, Faradiba Sarquis Serpa, Joaquin Sastre, Eleonora Savi, Agne Savonyte, Bisher Sawaf, Glenis K Scadding, Sophie Scheire, Peter Schmid-Grendelmeier, Juan Francisco Schuhl, Holger Schunemann, Maria Schvalbová, Jorgen Schwarze, Nicola Scichilone, Gianenrico Senna, Cecilia Sepúlveda, Elie Serrano, Sara Shamai, Aziz Sheikh, Mike Shields, Vasil Shishkov, Nikos Siafakas, Alexander Simeonov, Estelle FER Simons, Juan Carlos Sisul, Brigita Sitkauskiene, Ingelbjorg Skrindo, Tanja Soklič Košak, Dirceu Solé, Martin Sondermann, Talant Sooronbaev, Manuel Soto-Martinez, Manuel Soto-Quiros, Barnaro Sousa Pinto, Milan Sova, Michael Soyka, Krzysztof Specjalski, Annette Sperl, Otto Spranger, Sofia Stamataki, Lina Stefanaki, Cristiana Stellato, Rafael Stelmach, Timo Strandberg, Petra Stute, Abirami Subramaniam, Charlotte Suppli Ulrik, Michael Sutherland, Silvia Sylvestre, Aikaterini Syrigou, Luis Taborda Barata, Nadejda Takovska, Rachel Tan, Frances Tan, Vincent Tan, Ing Ping Tang, Masami Taniguchi, Line Tannert, Pongsakorn Tantilipikorn, Jessica Tattersall, Filippo Tesi, Uta Thieme, Carel Thijs, Mike Thomas, Teresa To, Ana Maria Todo-Bom, Alkis Togias, Peter-Valentin Tomazic, Vesna Tomic-Spiric, Sanna Toppila-Salmi, Maria-José Torres Jaen, Elina Toskala, Massimo Triggiani, Nadja Triller, Katja Triller, Ioanna Tsiligianni, M. Uberti, Ruxandra Ulmeanu, Jure Urbancic, Marilyn Urrutia Pereira, Martina Vachova, Felipe Valdés, Rudolf Valenta, Marylin Valentin Rostan, Antonio Valero, Arunas Valiulis, Mina Vallianatou, Erkka Valovirta, Michiel Van Eerd, Eric Van Ganse, Marianne van Hage, Olivier Vandenplas, Tuula Vasankari, Dafina Vassileva, Cesar Velasco Munoz, Maria Teresa Ventura, Cécilia Vera-Munoz, Frédéric Viart, Dilyana Vicheva, Pakit Vichyanond, Petra Vidgren, Giovanni Viegi, Claus Vogelmeier, Leena Von Hertzen, Theodoros Vontetsianos, Dimitris Vourdas, Vu Tran Thien Quan, Martin Wagenmann, Samantha Walker, Dana Wallace, Yun De Wang, Susan Waserman, Katrin Wehner, Magnus Wickman, Sian Williams, Dennis Williams, Nicola Wilson, Gary Wong, Kent Woo, Lucyna Wozniak, John Wright, Piotr Wroczynski, Paraskevi Xepapadaki, Plamen Yakovliev, Masao Yamaguchi, Kwok Yan, Yoke Yeow Yap, Mais Yassin, Barbara Yawn, Panayiotis Yiallouros, Arzu Yorgancioglu, Shigemi Yoshihara, Ian Young, Osman B Yusuf, Asghar Zaidi, Fares Zaitoun, Petra Zalud, Heather Zar, M. T. Zedda, Mario E Zernotti, Luo Zhang, Nanshan Zhong, Mihaela Zidarn, Torsten Zuberbier, Celia Zubrinich

**Affiliations:** 1 Department of Dermatology and Allergy, Charité, Universitätsmedizin Berlin, Humboldt-Universität Zu Berlin, Berlin Institute of Health, Comprehensive Allergy Center, Berlin, Germany; 2 University Hospital Montpellier, 273 avenue d’Occitanie, 34090 Montpellier, France; 3 MACVIA-France, Montpellier, France; 4 Laboratoire de Biochimie et Hormonologie, PhyMedExp, Université de Montpellier, INSERM, CNRS, CHU, Montpellier, France; 5 Medical Consulting Czarlewski, Levallois, France; 6 MASK-Air, Montpellier, France; 7 IMIM (Hospital del Mar Research Institute), Barcelona, Spain; 8 Universitat Pompeu Fabra (UPF), Barcelona, Spain; 9 CIBER Epidemiología y Salud Pública (CIBERESP), Barcelona, Spain; 10 ISGlobAL, Barcelona, Centre for Research in Environmental Epidemiology (CREAL), Barcelona, Spain; 11 Institute for Population Health Sciences, Barts and The London School of Medicine and Dentistry, Queen Mary University of London, London, UK; 12 Skin and Allergy Hospital, Helsinki University Hospital, and University of Helsinki, Helsinki, Finland; 13 GreenUPorto - Sustainable Agrifood Production Research Centre, DGAOT, Faculty of Sciences, University of Porto, Campus de Vairão, Vila do Conde, Portugal; 14 Department of Advanced Biomedical Sciences, Federico II University, Napoli, Italy; 15 Department of Geriatrics, Montpellier University Hospital, Montpellier, France; 16 Division of Allergy, Department of Pediatric Medicine, The Bambino Gesu Children’s Research Hospital Holy See, Rome, Italy; 17 Personalized Medicine Asthma and Allergy Clinic-Humanitas University & Research Hospital, IRCCS, Milano, Italy; 18 CINTESIS, Center for Research in Health Technology and Information Systems, Faculdade de Medicina da Universidade do Porto; and Medida,, Lda Porto, Porto, Portugal; 19 World Business Council for Sustainable Development (WBCSD) Maison de la Paix, Geneva, Switzerland; 20 AgroParisTech-Paris Institute of Technology for Life, Food and Environmental Sciences, Paris, France; 21 Microbiology and Functionality Research Group, Research and Development Division, World Institute of Kimchi, Gwangju, Korea; 22 SME Service Department, Strategy and Planning Division, World Institute of Kimchi, Gwangju, Korea; 23 Maladies Infectieuses et Tropicales, CHU, Montpellier, France; 24 The Usher Institute of Population Health Sciences and Informatics, The University of Edinburgh, Edinburgh, UK; 25 Swiss Institute of Allergy and Asthma Research (SIAF), University of Zurich, Davos, Switzerland

**Keywords:** COVID-19, Nrf2, Foods, Nutrients, Insulin resistance, Obesity, TRPA1

## Abstract

There are large between- and within-country variations in COVID-19 death rates. Some very low death rate settings such as Eastern Asia, Central Europe, the Balkans and Africa have a common feature of eating large quantities of fermented foods whose intake is associated with the activation of the Nrf2 (Nuclear factor (erythroid-derived 2)-like 2) anti-oxidant transcription factor. There are many Nrf2-interacting nutrients (berberine, curcumin, epigallocatechin gallate, genistein, quercetin, resveratrol, sulforaphane) that all act similarly to reduce insulin resistance, endothelial damage, lung injury and cytokine storm. They also act on the same mechanisms (mTOR: Mammalian target of rapamycin, PPARγ:Peroxisome proliferator-activated receptor, NFκB: Nuclear factor kappa B, ERK: Extracellular signal-regulated kinases and eIF2α:Elongation initiation factor 2α). They may as a result be important in mitigating the severity of COVID-19, acting through the endoplasmic reticulum stress or ACE-Angiotensin-II-AT_1_R axis (AT_1_R) pathway. Many Nrf2-interacting nutrients are also interacting with TRPA1 and/or TRPV1. Interestingly, geographical areas with very low COVID-19 mortality are those with the lowest prevalence of obesity (Sub-Saharan Africa and Asia). It is tempting to propose that Nrf2-interacting foods and nutrients can re-balance insulin resistance and have a significant effect on COVID-19 severity. It is therefore possible that the intake of these foods may restore an optimal natural balance for the Nrf2 pathway and may be of interest in the mitigation of COVID-19 severity.

## Introduction

Large differences in COVID-19 death rates exist between countries and regions of the same country. Like most diseases, COVID-19 exhibits large geographical variations which frequently remain unexplained. The COVID-19 epidemic is multifactorial, and factors like climate, population density, social distancing, age, phenotype, obesity and prevalence of non-communicable diseases are associated to increased incidence and mortality [1]. Diet represents only one of the possible causes of the COVID-19 epidemic [2, 3]. Although there are many pitfalls in analyzing death rates for COVID-19, [3] death rates were low or very low in Central European countries, Eastern Asian countries, many Sub-Saharan African countries, the Middle East, India and Pakistan as well as Australia and New Zealand. This geographical pattern is very unlikely to be totally due to reporting differences between countries. Some very low death rate settings (but not Australia or New Zealand) have a common feature of eating large quantities of fermented vegetables such as cabbage, other members of the Brassicaceae family and, in some continents, various spices [4]. Notwithstanding the fact that data from ecological studies need to be interpreted with caution, fermented vegetables or cabbage have been found to be associated with low COVID-19 death rates in European countries [5–7].

Reactive oxygen species (ROS) exert beneficial and toxic effects on cellular functions. Nrf2 is a pleiotropic transcription factor protecting against oxidative stress. It expresses a wide array of genes involved in immunity and inflammation, including antiviral actions [8]. Several Nrf2-interacting natural compounds (e.g. berberine, curcumin, epigallocatechin gallate, genistein, quercetin, resveratrol, sulforaphane) and lactobacilli acting as antioxidants are effective against insulin resistance associated diseases [9]. They may be important in the mitigation of COVID-19 [5, 9, 10], acting through the endoplasmic reticulum (ER) [11–13] or ACE-Angiotensin-II-AT_1_R axis (AT_1_R) pathway [3, 5] and leading to insulin resistance (IR), endothelial damage, lung injury and cytokine storm. They may also interact with SARS-CoV-2 by other pathways involved in IR that may be Nrf2-dependent or -independent [11–13].

Obesity is a very important risk factor for COVID-19 severity [14] and is often associated with diet. There may be interactions between obesity, diet and COVID-19, possibly linked with Nrf2 [15].

The present rostrum follows the first two papers on diet and COVID-19 from our group [3, 5]. Specifically, we seek to (i) expand discussion on the role of Nrf2-interacting natural nutrients in IR, (ii) assess the mechanisms on ER stress and the AT_1_R pathway, and (iii) understand how Nrf2-interacting nutrients can interplay to mitigate COVID-19.

### Nrf2-interacting nutrients

The most common Nrf2 nutrients include berberine, curcumin, epigallocatechin gallate (EGCG), genistein, quercetin, resveratrol, sulforaphane mostly found in vegetables and fruits, and *Lactobacillus* in fermented foods (Table 1). We did not want to be exhaustive and we did not examine other nutrients such as brassinin or the the organosulfide diallyl trisulfide.

**Table 1:** Origin of Nfr2-interacting nutrients

Nutrient		Foods containing nutrient
Berberine	Benzylisoquinoline alkaloid	European barberry, goldenseal, goldthread, Oregon grape, phellodendron, goldenseal, poppy, and tree turmeric
Curcumin	Curcuminoid (phenol)	Turmeric
EGCG	Catechin (polyphenol)	Green and white tea
Genistein	Soy isoflavone	Soy-based foods including tofu, tempeh and miso
*Lactobacillus*	Lactic acid bacteria	Fermented foods
Quercetin	Flavonoid group of polyphenols	Found in many fruits (cranberries, lingonberries, black plums), vegetables (broccoli, capers, kale, red onion, radish, sorel, watercress), leaves (fennel), seeds, and grains
Resveratrol	Stilbenoid (phenol)	Skin of grapes, blueberries, raspberries, mulberries and peanuts
Sulforaphane	Isothiocyanate	Cruciferous vegetables such as broccoli, Brussels sprouts, and cabbages

EGCG, Epigallocatechin gallate

Herbs, fruits or vegetables such as garlic [16] or kiwi can also have antioxidant activities mediated by Nrf2 [9].

Micronutrients such as Zinc, Chromium, Selenium [17] and vitamin D [18] possess antioxidant activities associated, at least partly, with activation of Nrf2.

### Cellular response to SARS-CoV-2

#### Endoplasmic reticulum stress response and Coronavirus

The coronavirus infection triggers ER stress responses in infected cells associated with increased levels of reactive oxygen species (ROS) and unfolded protein response (UPR) [19–21]. As a general response, ER stress leads to PERK phosphorylation of the elongation initiation factor 2α (eIF2α) and of Nrf2 [22]. Activated PERK inactivates eIF2α, leading to a decrease in overall protein synthesis. Phosphorylation of PKR and PERK has been observed in SARS-CoV-2-infected cells [23]. ERK/MAPK and PI3K/AKT/mTOR signalling responses play important roles in Middle East respiratory syndrome coronavirus (MERS-CoV) infection [24]. The key role in the synthesis of proteins essential for these mechanisms belongs to mTOR (mammalian target of rapamycin) complexes and signalling pathways involved in mTOR regulation including eIF2α [25]. mTOR is a serine/threonine protein kinase in the PI3K-related kinase (PIKK) family that forms the catalytic subunit of two distinct protein complexes, known as mTOR Complex 1 (mTORC1) and 2 (mTORC2). The mTOR pathway functions as a central regulator of cell metabolism, growth, proliferation, and survival. mTORC1 mainly functions as a nutrient/energy/redox sensor and controls protein synthesis, lipid metabolism, and organelle biogenesis [26]. mTORC2 promotes the activation of insulin receptors and insulin-like growth factor 1 receptors. mTORC1 and C2 complexes are activated by nutrients, growth factors, and inflammatory mediators.

ER stress and sustained UPR signalling are major contributors to the pathogenesis of several diseases, including inflammatory disorders and viral infections [27] and can increase the severity of these events [28]. ER stress has an important role in cardiovascular and metabolic disease, obesity and in diabetes [29, 30] and pancreatic ß-cell dysfunction, often through mTOR [31]. Oxidative stress is counter-balanced by complex antioxidant defence systems regulated by a series of multiple pathways, including the UPR, to ensure that the response to oxidants is adequate. Nrf2, interrelated with the UPR sensor called the pancreatic endoplasmic reticulum kinase, is a regulator of cellular resistance to oxidants [22, 32].

A recent study showed a disruption of mTOR signalling with increased levels of mTOR and a down-regulation of eIF2 signalling in multiple cellular compartments of severe COVID-19 patients when compared to patients who recovered [33].

### AT_1_R-associated effects

Angiotensin II (AngII) is the predominant Renin–Angiotensin–Aldosterone system (RAAS) component contributing to IR [34]. The angiotensin-converting enzyme 2 (ACE2) receptor is part of the dual RAAS system consisting of an AT_1_R axis and an ACE-2-Angiotensin-(1,7)-Mas axis. AT_1_R is involved in most of the effects of Ang II, including oxidative stress generation [35], which in turn upregulates AT_1_R [36]. In metabolic disorders and with older age, there is an upregulation of the AT_1_R axis leading to pro-inflammatory, pro-fibrotic effects in the respiratory system, endothelial damage and IR [37]. SARS-CoV-2 binds to its receptor ACE2 and exploits it for entry into the cell. The ACE2 downregulation, as a result of SARS-CoV-2 binding, enhances the AT_1_R axis [38] likely to be associated with IR [39, 40], but also with inflammation [41] and severe outcomes of COVID-19. Nrf2 is the most potent antioxidant in humans and can block the AT_1_R axis [8].

### Cross-talk between the renin-angiotensin-aldosterone system (RAAS) and the endoplasmic reticulum (Fig. 1)

**Fig. 1:**
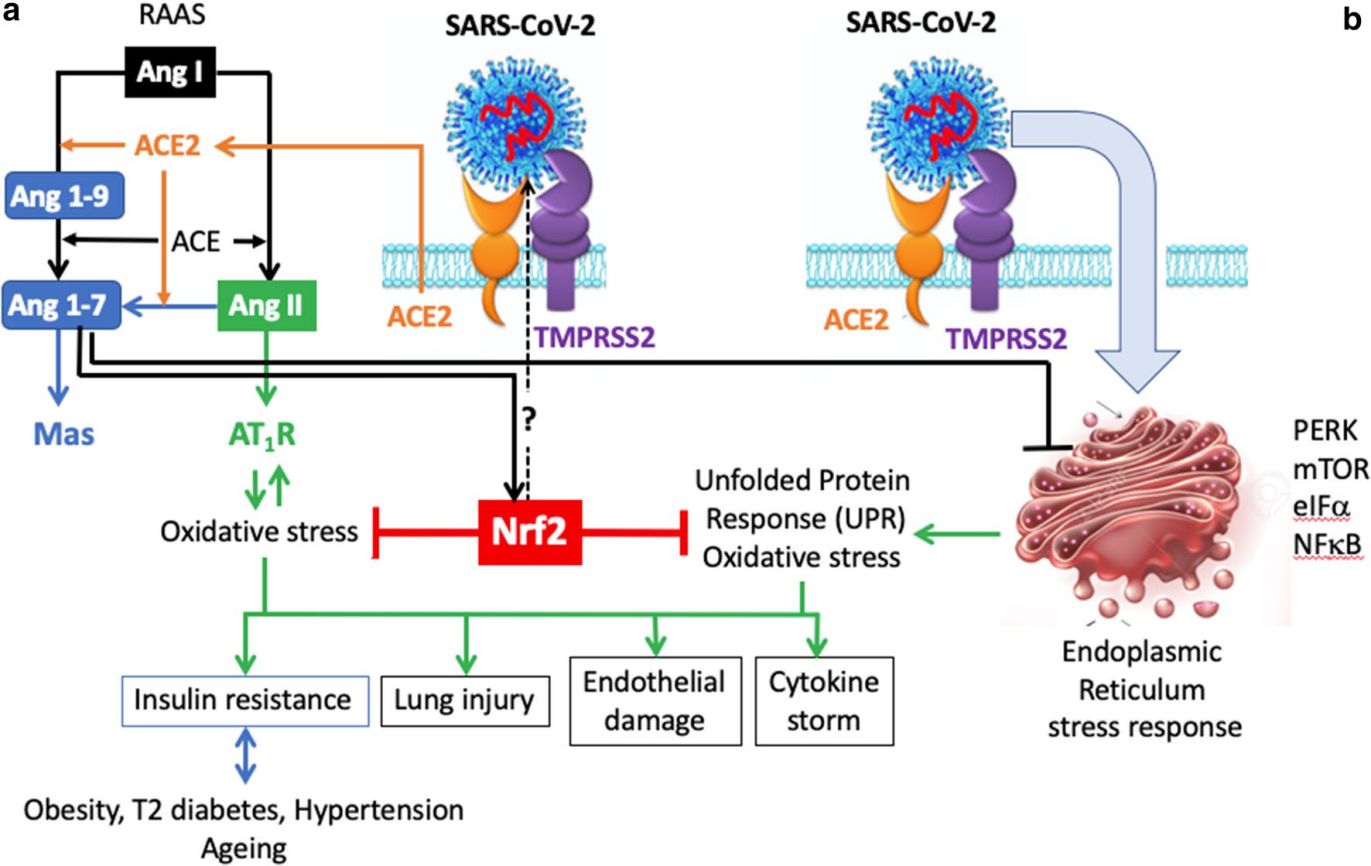
Interactions between the renin–angiotensin–aldosterone system and the endoplasmic reticulum in COVID-19 ( modified from 5)

Several studies have shown an interaction of RAAS and ER in insulin resistance. Ang-II increases ER stress in adipose tissue [42]. ACE2 regulates intramuscular fat by improving ER and mitochondrial function [43]. On the other hand, Ang 1–7 protects against Ang II-induced ER stress and endothelial dysfunction via the Mas receptor [44]. These mechanisms appear to be of great importance in COVID-19 and propose an interaction between ER stress and AT_1_R/Mas pathways with Nrf2 at the centre of the regulatory mechanism.

Moreover, in addition to reducing the production of infectious virions, the inhibition of ER glucosidases also impairs the entry of selected viruses via a post-receptor-binding mechanism [45].

### Nrf2 in cytokine storm, endothelium and lung damage

The Nrf2 signalling pathway regulates anti-inflammatory gene expression and inhibits the progression of inflammation [46]. In particular, the upregulation of Nrf2 signalling inhibits the overproduction of IL-6, pro-inflammatory cytokines, and chemokines as well as limiting the activation of NFĸB.

Failure to protect against oxidative stress-induced cellular damage leads to endothelial dysfunction in cardiovascular diseases and other pathologies associated with metabolic syndrome. Several antioxidant pathways are involved in cellular redox homeostasis, among which the Nrf2 signalling pathway is one of the most prominent [47].

Nrf2 induces cellular rescue pathways against oxidative pulmonary injury, abnormal inflammatory and immune responses, as well as apoptosis. The Nrf2 pathway can protect against various lung injuries including acute lung injury and acute respiratory distress syndrome [48].

### Autophagy

Autophagy is the natural cell regulated mechanism leading to the degradation of components through the action of the lysosomal system to remove unnecessary or dysfunctional components. It is a constitutive pathway upregulated under stressful conditions including oxidative stress, [49] ER stress or viral infection. One key element of viral infection is the fate of the virus in the cell.

While autophagy has been shown to act as an anti-viral defence, human viruses use multiple steps in endocytic and autophagy pathways to help viral propagation and escape immune response [50, 51]. Coronaviruses have adapted by producing many strategies to escape or to benefit via the inhibition and/or stimulation of autophagy [52]. SARS-Cov-2 most likely impacts autophagy by several mechanisms [52–55] including highjacking the autophagy machinery for their intracellular survival (canonical) [54] and expressing specific proteins to usurp components of the autophagy pathway and propagate in host cells (noncanonical) [52].

The oxidative stress associated with increases in reactive oxygen species (ROS) is interconnected with autophagy [56, 57]. Oxidative stress leads to oxidative damage of proteins, lipids, and nucleic acids. Autophagy is crucial in ROS generation and scavenging damaged substrates, which is achieved by the release and activation of Nrf2 [58]. A redox independent cross-talk also exists between the Nrf2-Keap 1 axis and autophagy through p62, an autophagy adaptor protein. p62 activates Nrf2 by a noncanonical pathway. p62 binds to Keap 1, the inhibitor of Nrf2, and induces Keap 1 degradation by autophagy [56]. Intermittent activation of Nrf2 through the canonical pathway confers cellular protection and functional integrity whereas prolonged activation of Nrf2 through the noncanonical pathway appears to be detrimental, resulting in tissue injuries and inflammation [49]. In acute lung injury, autophagy is induced by different stimuli including the oxidative stress [57]. However, the role of autophagy in acute lung injury still remains controversial depending on the underlying cause of the lung injury, on the cell types, and on the stage of lung injury. mTOR inhibition may be protective.

### Complexity of the anti-oxidant response

It is clear that Nrf2 is only one mechanism of the anti-oxidant stress and that multiple products can act on the anti-oxidant stress of COVID-19. As an example, sulforaphane protects against acetaminophen-induced hepatotoxicity [59]. Its anti-oxidant and anti-inflammatory activity may be enhanced in vitro by combining it with some medications used in COVID-19 such as acetaminophen [60]. Moreover, other mechanisms such as lipid rafts, autophagy, the fatty acid transporter CD36 and adipokines may play an equally important role.

### Nrf2-interacting nutrients and COVID-19

#### Interactions with COVID-19

Obesity, possibly hypertension, type 2 diabetes (T2D) and ageing all represent risk factors for severe COVID-19 associated with cytokine storm and IL-6, endothelial damage in different organs and lung damage.

IR is a pathological condition in which cells fail to respond normally to the hormone insulin. Major mechanisms of IR include oxidative stress, inflammation, insulin receptor mutations, endoplasmic reticulum stress, and mitochondrial dysfunction [61]. In COVID-19, IR can be induced by at least ER stress or the AT_1_R pathways. IR is a key component of the metabolic syndrome, a clustering of at least three of the five following medical conditions: abdominal obesity [62], high blood pressure [63], high blood sugar, high serum triglycerides, and low serum high-density lipoprotein (HDL) [64]. The metabolic syndrome is associated with the risk of developing cardiovascular disease and T2D [65, 66]. All nine Nrf2-interacting nutrients had some effect—although sometimes weak—against obesity, hypertension and T2D (Table 2).

**Table 2:** Effect of Nrf2-interacting nutrients on diseases associated with oxidative stress

	Insulin resistance						Lung injury	IL-6 Cytokines
	AT1R down regulation	Obesity	HTA	T2D	Endothelium damage	Ageing		
Berberine		[161, 162]	[162]	[162, 173, 174]	[175]	[176]	[173]	[173]
Curcumin	[105]	[163, 164]	[177]	[178]	[179]	[180]	[181, 182]	[183]
EGCG	[106]	[164]	[184]	[185]	[184]	[180]	[186, 187]	[188]
Genistein	[107, 108]	[165]	[189]	[190]	[189]	[180]	[191]	[192]
*Lactobacillus*		[168, 169]	[193]	[194]	[195]	[196]	[197]	[198]
Quercetin		[163]	[199]	[200]	[184]	[180]	[201]	[202]
Resveratrol	[109]	[163, 164]	[203]	[204]	[205]	[180]	[206]	[207]
Sulforaphane		[170]	[208]	[208]	[209]	[180]	[210]	[211]

EGCG: Epigallocatechin gallate

Search strategy: For this table, in order to compare the mechanisms of action and properties of Nrf2-interacting nutrients, a PubMed search was initiated. This was not a systematic review, but an attempt to assess whether the impact on the disease has been described

We searched PubMed using the display option “best matches”

We first searched “systematic reviews” by PubMed for the different nutrients and we collected the first “best match” systematic review related to the question

If there was no systematic review, we searched for “reviews” and we collected the first “best match” review related to the question

If there was no review, we searched for papers and we collected the first “best match” paper related to the question

IR is frequently associated with endothelial dysfunction and has been proposed to play a major role in cardiovascular [67], kidney [68] or cerebrovascular diseases [69]. All nine Nrf2-interacting nutrients had an effect against endothelial damage.

Ageing is associated with IR [70] and all nine Nrf2-interacting nutrients had an effect on ageing. All nine Nrf2-interacting nutrients reduce IL-6 and cytokines.

Most Nrf2-interacting nutrients have an action on mTOR, PPARγ, NFκB, ERK and eIF2α (Table 3).

**Table 3:** Mechanisms involved in the antioxidant effects of Nrf2-interacting nutrients

	Nrf2	mTOR	PPARγ	NFκB	ERK	eIF2α
Effect	Activation	Inhibition	Activation	Inhibition	Activation	Inhibition
Berberine	[173]	[176]	[212]	[71]	[176]	[87]
Curcumin	[180, 213]	[213, 214]	[213]	[213]	[213]	[88]
EGCG	[180]	[214]	[215]	[216, 217]	[217]	[90]
Genistein	[180]	[218]	[219]	[220] [221]	[222]	[91]
*Lactobacillus*	[223]	[224]	[225]	[223]	[226]	
Quercetin	[180]	[227]	[219]	[228]	[229]	[92]
Resveratrol	[180]	[214, 230]	[219]	[220]	[109]	[93]
Sulforaphane	[180]	[98, 231]	[208]	[208]	[232]	[233]

EGCG: Epigallocatechin gallate

The search strategy used in Table 2 was applied in an attempt to assess whether a mechanism of action could be identified

### Anti-viral effects

Nrf2-interacting nutrients have large antiviral activities demonstrated in humans and animals (Table 4).

**Table 4:** Antiviral effects of Nrf2-interacting nutrients

	Antiviral	COVID	STING
Berberine	[71]	[71]	
Curcumin	[72]	[234–237]	
EGCG	[238]	[239–242]	[243]
Genistein	[244]	[245]	
*Lactobacillus*	[246]	[246, 247]	
Quercetin	[248]	[249–253]	
Resveratrol	[254]	[255–259]	
Sulforaphane	[260]		[84]

The search strategy used in Table 2 was applied in an attempt to assess whether anti-viral or anti-COVID properties have been described

Berberine through NFκB and MAPK pathways has an anti-viral activity on several viruses, and potentially against SARS-CoV-2 [71]. Curcumin can block the entry of viruses into cells or its replication in the cell [72]. It acts on NFκB [73] or MAPK [74]. EGCG has multiple antiviral properties possibly though MAPK [75].

The suppressive effects of EGCG on viral replication were abolished in cells with knocked-down Nrf2 expression [76]. siRNA-mediated depletion of Nrf2 boosted HIV infectivity in primary macrophages and reduced the anti-viral effects of sulforaphane [77]. In a murine model, RSV-induced bronchopulmonary inflammation, epithelial injury, and mucus cell metaplasia as well as nasal epithelial injury were significantly greater in Nrf2(-/-) mice than in Nrf2(+ / +) mice. Sulforaphane pre-treatment significantly limited lung RSV replication and virus-induced inflammation in Nrf2(+ / +) but not in Nrf2(-/-) mice. This effect may be mediated though NFκB [78]. Sulforaphane through Nrf2 significantly suppressed the hepatitis C virus (HCV) protein and RNA levels in HCV replicon cells and infectious system [79]. Caffeic acid could modulate Keap1/Nrf2 interaction via increasing p62 expression, leading to the stabilization of Nrf2 and HO-1 induction, and an elicit IFNα antiviral response to suppress HCV replication [80]. HCV genome replication was also suppressed in HCV sub-genomic replicon-bearing cells by bardoxolone methyl (BARD), an Nrf2 activator [81].

Type I IFNs (IFNα and -β) are central to immune-protection against viral infection [82]. A balanced production of type I IFNs is needed for the protection against virus, but excessive production is a potent driver of pathology [82]. Intracellular DNA and RNA sensors are essential in the innate immune response to viruses, causing the secretion of type I IFNs, cytokines and chemokines from infected cells. Viral cytosolic DNA is recognized by DNA sensors such as cyclic GMP-AMP synthase (cGAS) and its downstream signalling effector stimulator of interferon genes (STING) [83]. Sulforaphane through Nrf2 activation decreases STING expression and responsiveness to STING agonists while increasing susceptibility to infection with DNA viruses [84]. Reduction of STING expression by Nrf2 is mechanistically distinct from how Nrf2 reduces the release of the pro-inflammatory cytokines IL-1β and IL-6 [84]. Nrf2 negatively regulates Type I INF responses and increases susceptibility to herpes genital infection in mice [85]. Itaconate is a crucial anti-inflammatory metabolite that acts via Nrf2 to limit inflammation and modulate type I IFNs [86].

### mTOR and eIF2α

Several Nrf2-interacting nutrients act through mTOR or eIF2α. The insulin-sensitizing action of berberine was related to reducing ER stress in Hep G2 cells. The levels of phosphorylation both on PERK and eIF2α were inhibited in cells pretreated with berberine [87]. In an IR animal model, curcumin was found to act on eIF2α [88]. The induction of the ER stress pathway by green tea EGCG in colorectal cancer cells is mediated by the activation of PERK [89]. The proteasome inhibitors Bortezomib (BZM) and MG132 trigger cancer cell death via induction of ER stress and UPR. EGCG antagonizes BZM toxicity by exacerbating the activation of autophagy and eIF2α up-regulation [90]. In rats, genistein protects against acute pancreatitis via the activation of an apoptotic pathway mediated through activation of multiple ER stress-related regulators like GRP78, PERK, and eIF2α [91]. Quercetin blocks airway epithelial cell chemokine expression though eIF2α phosphorylation [92]. Pterostilbene (PT), a natural analogue of resveratrol, inhibits hepatocellular cell (HCC) growth without the induction of apoptosis in an ER stress- and autophagy-dependent manner through the eIF2α pathway [93]. Resveratrol modulates response against acute inflammatory stimuli in aged mouse brain. ER stress markers demonstrated significant changes in resveratrol-treated mice after LPS treatment, specifically in eIF2α [94]. Other studies have found an effect of resveratrol on eIF2α [95, 96].

Sulforaphane exerts a neuroprotective effect involving Nrf2-dependent reductions in oxidative stress, mTOR-dependent inhibition of neuronal apoptosis, and the restoration of normal autophagy [97]. Sulforaphane also inhibits mTOR in an Nrf2-independent manner [98].

Kimchi attenuates fatty streak formation in the aorta of low-density lipoprotein receptor knockout mice via the inhibition of ER stress (via several mechanisms including eIF2α) and apoptosis [99]. Nutrients originating from Kimchi and its ingredients modulate the Nrf2/PERK signalling pathway to homeostasis in oxidative stress states. Kimchi and its bioactive compound ((3–4′-hydroxyl-3′,5′-dimethoxyphenyl) propionic acid: HDMPPA), which is a metabolite result from fermentation, alleviate oxidative stress and inflammatory response not only via the Nrf2 pathway, but also via the PERK/CHOP pathway, which induced apoptosis of ER, in cardiovascular disease and ageing models [100–102]. In addition, Arvelexin from *Brassica rapa* and anthocyanin-rich extract from red cabbage exert anti-inflammatory properties by the inhibition of NF- κB activation and by Nrf2-regulated HO-1 induction in macrophages and apolipoprotein E-deficient mice [103, 104], suggesting that Nrf2 activation during inflammation antagonizes the NF-κB pathway. It is possible that the intake of Kimchi may help to mitigate COVID-19 outcomes by maintaining or restoring the Nrf2 system.

### AT_1_R

Curcumin [105], EGCG [106], genistein [107, 108] and resveratrol [109] impact the AT_1_R pathway. NADPH oxidases of the Nox family are important sources of ROS and important agents in hypertension. They increase blood pressure in the presence of Ang II, an important and potent regulator of cardiovascular NADPH oxidase, via AT_1_R. Several natural compounds such as berberine, curcumin, quercitine, resveratrol and others are Nox inhibitors [110]. Dietary curcumin supplementation can increase antioxidant activity through the induction of heme oxygenase-1, a scavenger of free radicals, and through the reduction of reactive oxygen species and Nox-2 [111]. Sulforaphane reduces Ang II-induced vascular smooth muscle cells through Nrf2 signalling [112].

### mTOR and autophagy

The autophagic process is initiated by inactivation of the mechanistic/mammalian target of rapamycin (mTOR), the major autophagy suppressor [52]. The role of mTOR is unclear in coronavirus infection [52]. Nrf2 can directly regulate mTOR [113]. Certain mTOR or Rac1 inhibitors derived from rapamycin and azathioprine activate autophagy [51]. mTOR inhibitors were proposed to be tested in COVID-19 [114]. Many Nrf2-interacting nutrients are mTOR inhibitors and might have a role in autophagy.

### TRPA1 and TRPV1

Several Nrf2-interacting nutrients are direct TRPA1 (transient receptor potential ankyrin 1) [115] or TRPV1 (transient receptor potential vanilloid 1) activators. TRPA1 induces inflammation, plays key roles in the physiology of almost all organs and exhibits a high sensitivity of TRPs to oxidants. It is involved in many COVID-19 symptoms. TRPA1 can be activated by many foods (Table 5). There is a substantial overlapping of electrophilic ligands between TRPA1 and Nrf2. It has been suggested that the two systems might be part of the same network, with TRPA1 representing the sensory arm, and Nrf2 its biochemical counterpart [115]. However, not all Nrf2-interacting nutrients are activators of TRPA1 and mustard oil, the first TRPA1 agonist found [116], does not interact with Nrf2.

**Table 5:** TRPA1 and TRPV1 interactions of Nrf2-interacting nutrients

	TRPA1	TRPV1
Berberine		Antagonist [261]
Curcumin	Antagonist [262]	Antagonist [262]
EGCG	Agonist [263]	Antagonist [264]
Genistein	Antagonist [265]	Antagonist [266]
*Lactobacillus*		Antagonist
Quercetin	Agonist [267]	Antagonist [268]
Resveratrol	Antagonist [269, 270]	Antagonist [271]
Wasabi	Agonist [272]	Agonist [272]
Capsaicin	Agonist [273]	Agonist [274]

The search strategy used in Table 2 was applied in an attempt to assess whether a mechanism of action could be identified

In COVID-19, some Nrf2-interacting nutrients may act by desensitizing TRPA1 (and possibly TRPV1) receptors (Bousquet et al. in preparation).

### Complex interactions in oxidative stress

**Fig. 2:**
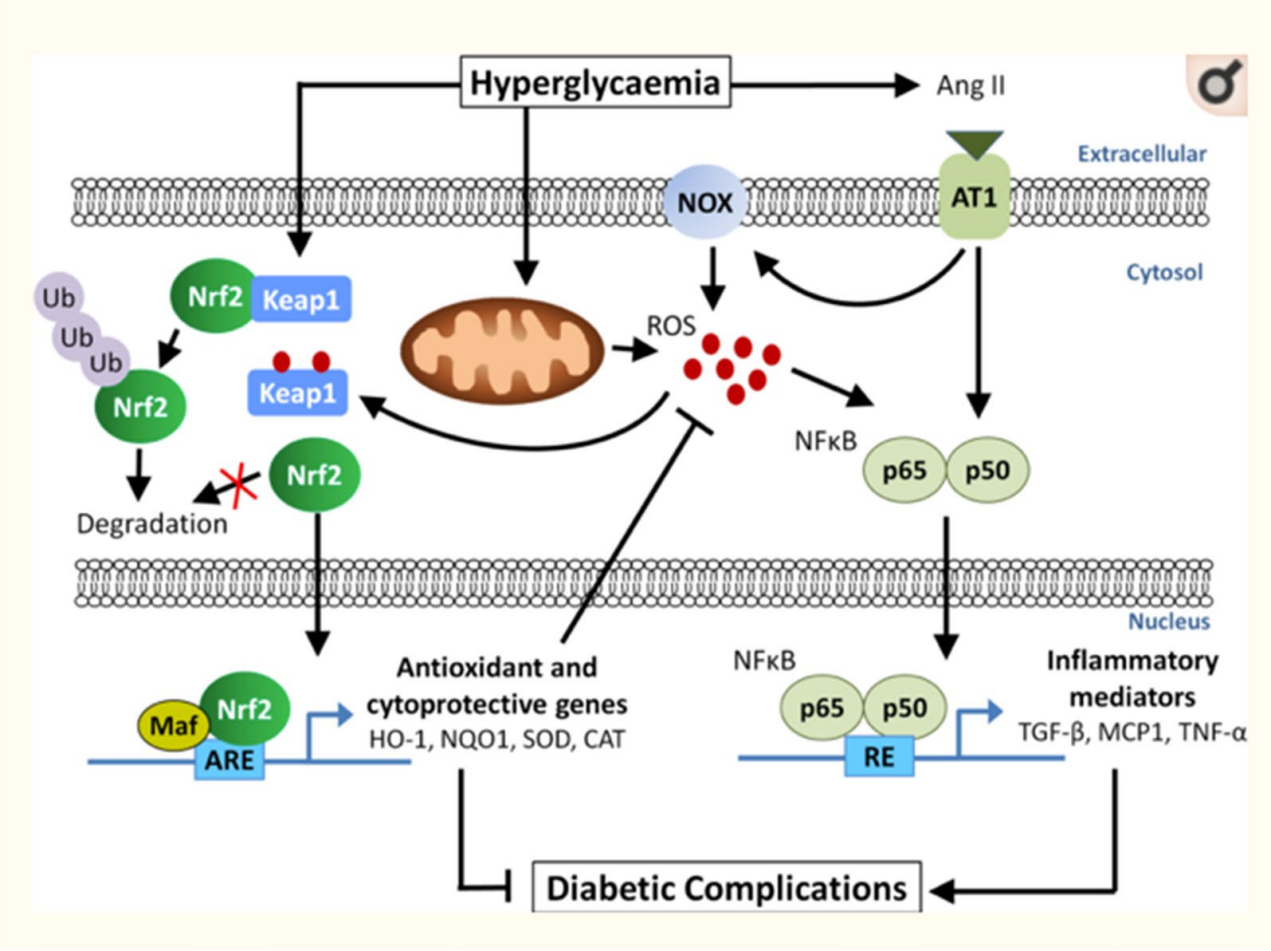
Complex interactions leading to oxidative stress in diabetes (from [117])

IR induces oxidative stress either through the overproduction of superoxide by ER stress or the activation of Ang II-mediated upregulation of nicotinamide adenine dinucleotide phosphate (NADPH)-oxidase (NOX) activity, resulting in the cytosolic production of ROS [117] (Fig. 2).

One of the key features of the complex interaction between nutrients and the oxidative stress/inflammatory response is the differential regulation of NFκB and Nrf2 by the cell redox status [118]. Nrf2 and NFκb are present in an inactive form in the cytosol since they are linked to an inhibitory compound iNFκB or INrf2 (Keap 1), both targets of reactive oxygen species [119–121]. In the case of a large production of ROS, which would overwhelm the antioxidant defence, iNFκB is oxidized and catabolized. Furthermore, NFκB is translocated to the nucleus and initiates the expression of inflammatory proteins such as cytokines, chemokines, adhesion molecules, cytokine receptors, iNO synthases, lipoxygenases, cyclooxygenases and growth factors [122, 123]. Once produced, cytokines are able to activate oxidant production by the NADPH oxidase complex, leading to an oxidative burst, which could in turn enhance NFκB activation. Thus, NFκB activation results in a directional and synergistic linkage of inflammation and oxidative stress [120, 124].

The canonical pathway of Nrf2 activation also involves changes in the cell redox state [189]. A weak or controlled ROS production results in the degradation of Keap 1. Thus, Nrf-2 could be translocated to the nucleus, binds to the antioxidant response element and activates an antioxidant enzyme such as Heme Oxigenase, SOD and catalase or cytoprotective genes [125, 126]. It could also reduce the production of ROS [127]. The increase in antioxidant defence maintains or restores the cellular redox state. In addition, Nrf2 stimulation could downregulate NFkB activation [128, 129]. In fact, redox signalling appears as a black box, controlling both NrF2 and NfκB activation and thus regulating inflammation and reparation. It is now recognized that the regulation of both pathways, NfκB and Nrf2, in part linked to the redox status, involved a cross talk to bring a coordinated inflammatory response [130, 131]. The intensity of the ROS insult could be a key factor in the imbalance of the NFκB/Nrf2 system [132]. In the case of oxidative stress, stimulation of NFκB (associated with a degradation of both Keap 1 and Nrf2) results in an amplification loop of inflammation. Thus, an imbalance between the NFκB and Nrf2 pathways has already been observed in T2D [112] or in multiple sclerosis. By contrast, an active and effective anti-oxidant system could result in a preventive loop leading to anti-oxidative and anti-inflammatory response. In this context, a positive modulation of Nrf2 by nutrients could act as an «oxidative pre-conditioning» system, and the resulting increase in the antioxidant enzyme could attenuate ROS deleterious effects and maintain cell integrity [133, 134].

This black box redox system could be effective in respiratory infection, particularly in COVID-19 [122]. Indeed, COVID-19 activates RAAS and induces ER stress, resulting in ROS production [32, 33], which could be further enhanced by risk factors such as obesity, diabetes, and hypertension [135–137]. Interestingly, RAAS activation seems related to COVID infection severity [41]. If the ROS production overwhelms antioxidant defence, a vicious circle linking oxidative stress and inflammation is initiated leading to a cytokine storm, as well as lung and endothelial injury. On the other hand, if Nrf2 is activated via nutrients, the antioxidant response could maintain or restore an adequate redox status. This would lead to an antioxidant and anti-inflammatory response resulting in a pauci-symptomatic infection. Interestingly, very recently, a similar effect on the Nrf2/NfkB balance via redox signalling was hypothesized via ozone therapy [138].

However, although the therapeutic potential of Nrf2 raised great hopes in the early 2010s [139], Nrf2 levels vary significantly depending on the physiological and pathological context. Thus, a properly timed and targeted manipulation of the Nrf2 pathway is critical for an effective treatment [140]. Surprisingly, only one Nrf2-based treatment has been approved: dimethyl fumarate [141], not devoid of side effects [142, 143]. This suggests that the balance is difficult to reach in drug development. Nrf2 overexpression may also be associated with diabetic nephropathy or retinopathy [117]. Recently, well-designed clinical trials with bardoxolone, an Nrf2 antagonist, were cancelled or stopped due to safety concerns [144]. The Nrf2 system plays an important role in the body's natural defence against hyperglycaemia-induced damage. However, this initial adaptive response to counteract the diabetes-driven oxidative stress appears to be short-lived, after which the Nrf2 system becomes overwhelmed under chronic glucose stimulation [117].

### Obesity, diet, Nrf2 and COVID-19

**Fig. 3:**
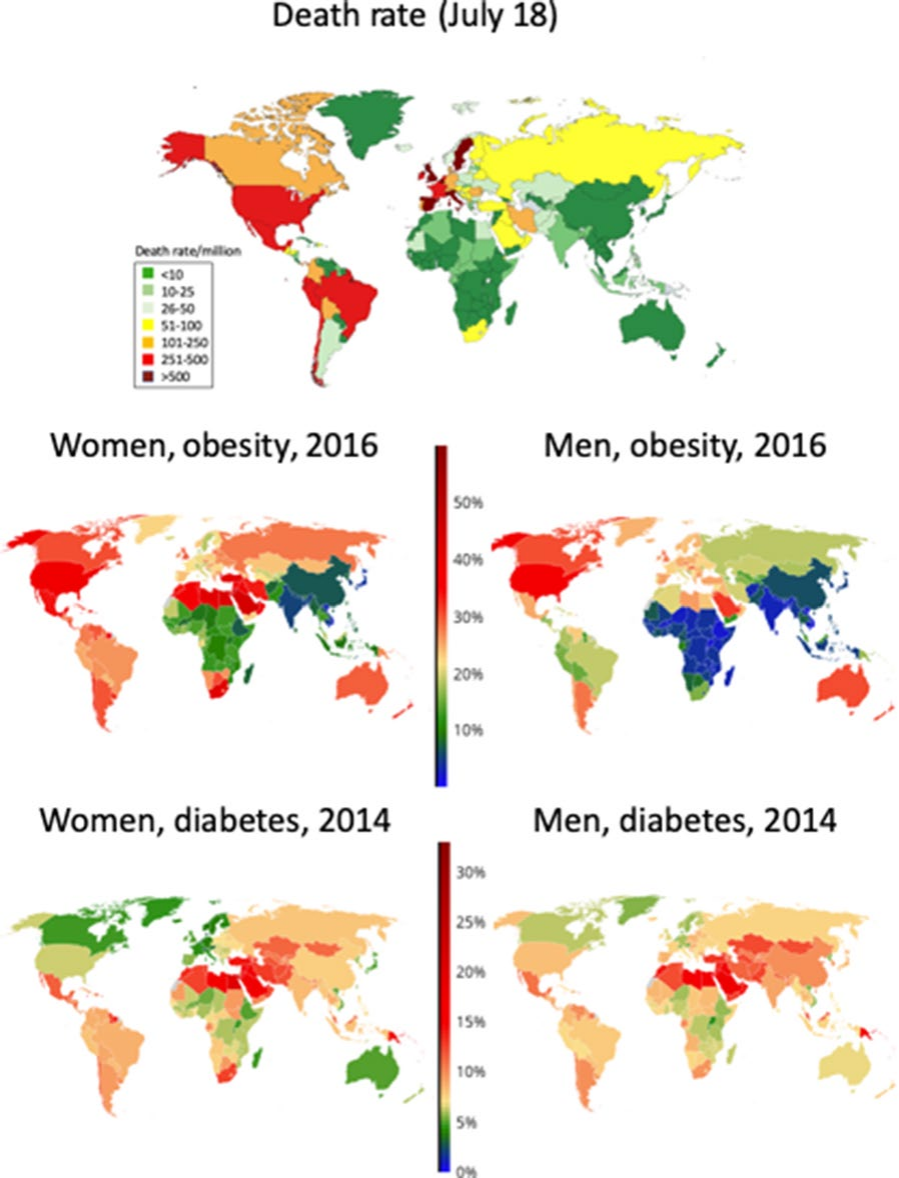
Prevalence of obesity, diabetes (NCD Risk Factor Collaboration (NCD-RisC, http://ncdrisc.org) and the COVID-19 death rate (Johns Hopkins Coronavirus Resource Center, https://coronavirus.jhu.edu)

In general, T2D and obesity prevalence are associated and the following has been stated by the NCD Risk Factor Collaboration (NCD-RisC) “The upsurge of T2D reflects the global obesity epidemic” [145]. However, many countries in Sub-Saharan Africa or Eastern Asia have a very low obesity prevalence that is not necessarily associated with a low diabetes prevalence (Fig. 3). These countries have the lowest obesity prevalence as well as the lowest COVID-19 death rates. Obesity is lower in Canada than in the US and this may partly explain differences in COVID-19 severity between these two countries. Obesity is high in South Africa, possibly explaining the higher death rate in this country than in other Sub-Saharan African countries.

**Fig. 4:**
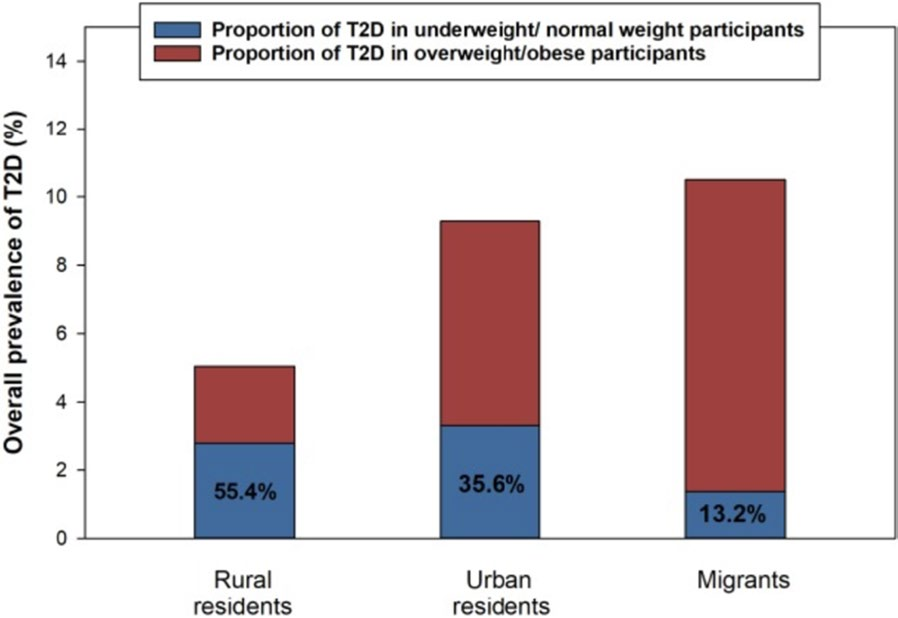
Links between obesity and diabetes in Ghanaians [150]

Many factors can explain this diabetes/obesity paradox. Genetic differences between countries are clear. However, the RODAM (Research on Obesity & Diabetes among African Migrants) study used a unique approach of comparing Ghanaians resident in the Netherlands, Germany, UK and Ghana to unravel the causes of obesity and T2D among African migrants and non-migrants. It showed striking differences suggesting that environmental factors are of great importance. Globally, one in 10 individuals is affected by T2D. In migrants, there is a higher T2D prevalence, the age of onset is younger and complications are more severe. One of the main determinants of T2D is obesity, which also disproportionally affects migrants [146–149]. In rural Ghanaians, most T2D is independent of obesity [150] (Fig. 4). Differences in food preferences were found across study sites: (i) in rural Ghana, diet concentrated on starchy foods (“roots, tubers, and plantain” diet) including cassava, (ii) in urban Ghana, nutrition was dominated by animal-based products, and (iii) in Europe, diet was highly diverse [151]. The “roots, tubers, and plantain” diet was directly associated with increased 10-year cardiovascular disease risk [152] but the relationship between diet and T2D was unclear [153]. In the national Korean cohort, obesity (50.4%) and abdominal obesity (47.8%) are associated with diabetes [154].

In COVID-19, obesity is a more severe risk factor than T2D [155]. There is a dose-dependent association of obesity with worse COVID-19 morbidity requiring hospitalization and intensive care and with mortality. This particularly applies to patients younger than 50 to 60 years of age [156]. Obesity is an important independent risk factor for serious COVID-19 disease [157, 158]. The association between BMI and COVID-19-related mortality was U-shaped, both in type 1 diabetes and in T2D (lowest risk for those with a BMI of 25·0–29·9 kg/m^2^) [159]. These data suggest differences between these two features of the metabolic syndrome for COVID-19 severity.

Nrf2 is also involved in complications of Type-1 diabetes [160]. All nine Nrf2-interacting nutrients had an effect against obesity, often through IR [161–170] (Table 2). In addition, Nrf2 may improve adipogenesis and adipocyte differentiation [171]. Thus, diet may be important in the prevention/management of obesity and, at the same time, may reduce the impact of COVID-19.

## Conclusions

**Fig. 5:**
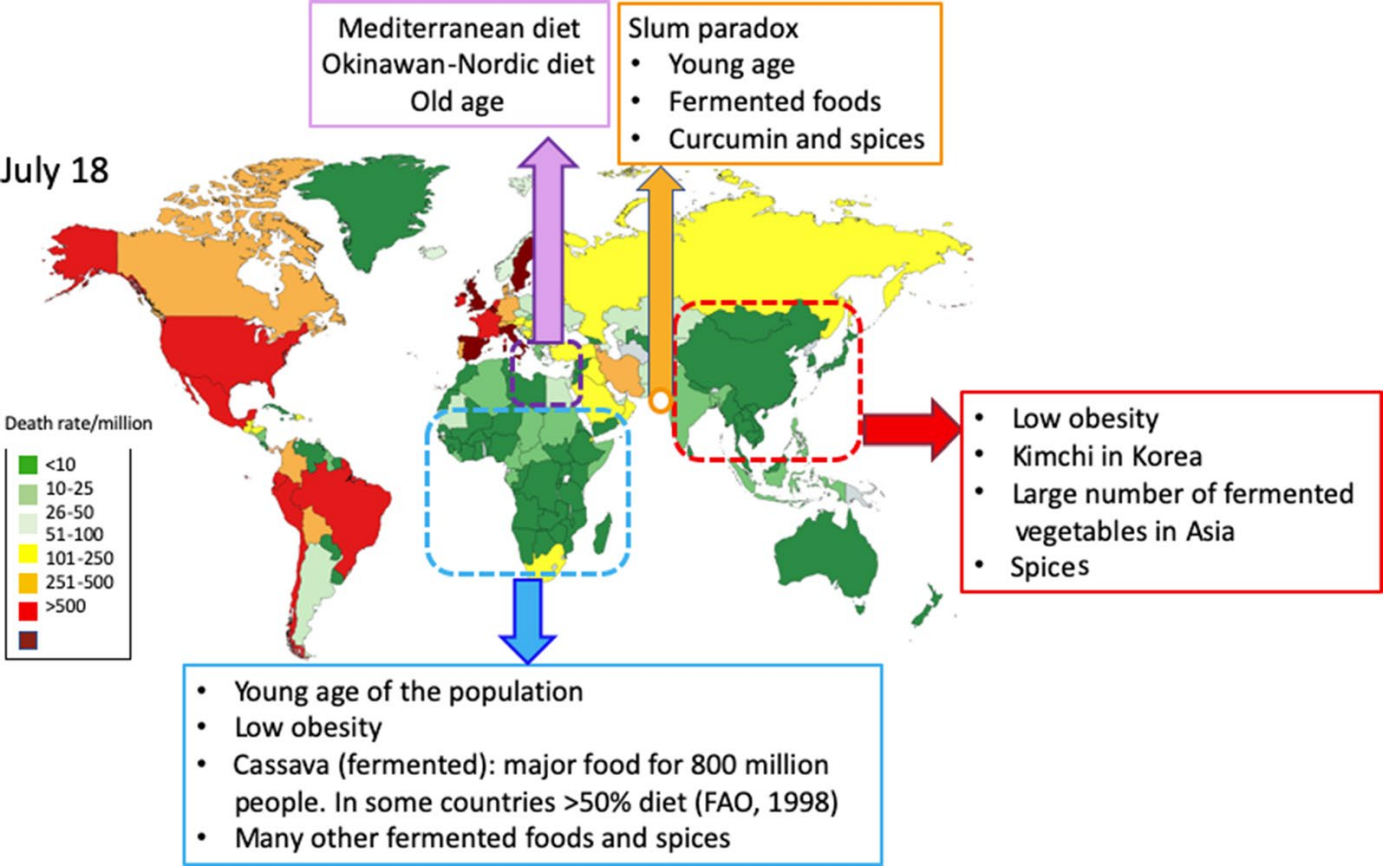
Geographic differences in COVID-19 death rates (Johns Hopkins Coronavirus center) and diet possibly explaining these differences

Interestingly, all nutrients tested had a similar effect on IR, cytokine storm, lung injury and endothelial damage. They were all active on most of the tested Nrf2 pathways. These data strongly suggest a common mechanism of action for all nutrients. These effects appear to be highly conserved [172]. However, we need to understand the differences between obesity and T2D in some countries with low obesity prevalence. These mechanisms may help to better appraise the potential severity of COVID-19 (Fig. 5).

It is tempting to propose that Nrf2-interacting foods and nutrients can help re-balance IR, and that they can have a significant effect on COVID-19 severity, and possibly also on susceptibility to infection by SARS-CoV-2. It is therefore possible that an increasing intake of specific foods may achieve an optimal natural balance for the Nrf2 pathway, since COVID-19 death rates, used as a proxy of severity, are low or very low in some countries where Nrf2-interacting nutrients are largely used (Fig. 5). Understanding the balance between Nrf2-interacting foods and nutrients would help to: (i) better understand the mechanisms of the oxidative stress in the IR diseases, (ii) develop optimal Nrf2-interacting nutrients and diets to reduce the prevalence and severity of IR diseases, (iii) optimize Nrf2 drug development and (iv) develop these strategies to mitigate COVID-19 severity.

There are still many unresolved questions requesting research on the time of onset of any efficacy of foods in COVID-19, the amount of the food to be administered and the interactions with the microbiome.

## Data Availability

Not applicable.

## Abbreviations

ACE: Angiotensin converting enzyme
AKT: Protein kinase B Ang II: Angiotensin II
AT_1_R: Angiotensin II receptor type 1
COVID-19: Coronavirus 19 disease
DNA: Desoxyribonucleic acid
EGCG: Epigallocatechin gallate
eIF2a: Elongation initiation factor 2α
ER: Endoplasmic reticulum
ERK: Extracellular signal-regulated kinases
GI: Gastro-intestinal
HCV: Hepatitis C virus
HIV: Human immunodeficiency virus
IFN: Interferon
IR: Insulin resistance
Keap1: Kelch-like ECH-associated protein 1
LAB: Lactic acid bacilli
mTOR: Mammalian target of rapamycin
mTORC: MTOR complex
MAPK: Mitogen-activated protein kinases
NADPH: Nicotinamide adenine dinucleotide phosphate
NF-κB: Nuclear factor kappa B
Nox: NADPH oxydase
Nrf2: Nuclear factor (erythroid-derived 2)-like 2
PI3K: Phosphoinositide 3-kinase
PPAR: Peroxisome proliferator-activated receptor
PERK: Protein kinase R (PKR)-like endoplasmic reticulum kinase
PKR: Protein kinase R
RAAS: Renin–Angiotensin–Aldosterone system
ROS: Reactive oxygen species
RSV: Respiratory syncytial virus
SARS: Severe acute respiratory syndrome
SARS-Cov-2: Severe acute respiratory syndrome coronavirus 2
STING: Signalling effector stimulator of interferon genes
TRPA1: Transient receptor potential ankyrin 1
TRPV1: Transient receptor potential vanillin 1
T2D: Type 2 diabetes
UPR: Unfolded protein response
